# Fully automated dose prediction using generative adversarial networks in prostate cancer patients

**DOI:** 10.1371/journal.pone.0232697

**Published:** 2020-05-04

**Authors:** Yu Murakami, Taiki Magome, Kazuki Matsumoto, Tomoharu Sato, Yasuo Yoshioka, Masahiko Oguchi

**Affiliations:** 1 Graduate Division of Health Sciences, Komazawa University, Komazawa, Setagaya-ku, Tokyo, Japan; 2 Radiation Oncology Department, Cancer Institute Hospital, Japanese Foundation for Cancer Research, Ariake, Koto-ku, Tokyo, Japan; St. Vincent Medical Center, UNITED STATES

## Abstract

**Purpose:**

Although dose prediction for intensity modulated radiation therapy (IMRT) has been accomplished by a deep learning approach, delineation of some structures is needed for the prediction. We sought to develop a fully automated dose-generation framework for IMRT of prostate cancer by entering the patient CT datasets without the contour information into a generative adversarial network (GAN) and to compare its prediction performance to a conventional prediction model trained from patient contours.

**Methods:**

We propose a synthetic approach to translate patient CT datasets into a dose distribution for IMRT. The framework requires only paired-images, i.e., patient CT images and corresponding RT-doses. The model was trained from 81 IMRT plans of prostate cancer patients, and then produced the dose distribution for 9 test cases. To compare its prediction performance to that of another trained model, we created a model trained from structure images. Dosimetric parameters for the planning target volume (PTV) and organs at risk (OARs) were calculated from the generated and original dose distributions, and mean differences of dosimetric parameters were compared between the CT-based model and the structure-based model.

**Results:**

The mean differences of all dosimetric parameters except for D_98%_ and D_95%_ for PTV were within approximately 2% and 3% of the prescription dose for OARs in the CT-based model, while the differences in the structure-based model were within approximately 1% for PTV and approximately 2% for OARs, with a mean prediction time of 5 seconds per patient.

**Conclusions:**

Accurate and rapid dose prediction was achieved by the learning of patient CT datasets by a GAN-based framework. The CT-based dose prediction could reduce the time required for both the iterative optimization process and the structure contouring, allowing physicians and dosimetrists to focus their expertise on more challenging cases.

## Introduction

Over the last few decades, it has become possible to adapt intensity modulated radiation therapy (IMRT) and volumetric modulated radiation therapy (VMAT) for almost all treatment sites. Owing to the complex dose distributions in IMRT and VMAT, radiation doses to normal tissues such as organs at risk (OARs) can often be significantly decreased even when the OARs are adjacent to the target, which reduces the risk of adverse events after radiotherapy [1]. However, these unique dose distributions have led to increasingly complex treatment planning procedures for IMRT and VMAT. It is very time consuming to delineate numerous structures, including the optimization-specific regions of interest, for the optimization of IMRT, and to repeat the optimization processes, including the tuning of dose constraint parameters, in order to achieve the desired dose distribution [2]. These arduous tasks force the dosimetrist and physician to devote a great deal of attention to the treatment planning. In addition, the large amount of time required for the treatment plan can lead to delays in the start of treatment. Such treatment postponement can influence tumor growth [3], and can lead to the misregistration of tumor localization and difficulties in immobilization methods, such as those using a vacuum pillow or thermoplastic shell, in daily treatments.

In the fairly recent past, researchers have begun to use of deep neural networks (DNN) to predict the dose distribution, engendering a new field of research [4–12]. Such dose prediction is useful for confirming the achievable dose distribution before or during the creation of treatment planning, and could reduce the iterative optimization process for IMRT, because the treatment planner can know which areas should receive increased or reduced doses based on the results of the prediction. Nguyen et al. reported that U-net-based architecture enabled prediction of the dose distribution in prostate cancer patients, and the average value of the absolute differences between the original and predicted dose was found to be less than 5% of the prescription dose [4]. Mahmood et al. predicted the dose distribution of simultaneous-integrated boost (SIB) for oropharyngeal cancer patients using a generative adversarial networks (GAN) framework, and compared the prediction performance of their GAN-based approach to several state-of-the-art techniques. They found that the GAN outperformed the U-net-based prediction model in terms of satisfying the clinical criteria, and the GAN also had the best overall performance among the methods examined [5].

Although the DNN-based prediction models achieve good agreement between the predicted and original dose distributions, patient contours are necessary for the prediction in all the frameworks [4–12]. Structure contouring can be highly time-consuming: for example, an average time of approximately 4 hours is needed to contour for prostate treatment planning with Eclipse (Varian Medical Systems, Palo Alto, CA), and contouring for patients with head and neck cancer can take much longer [2]. A significant portion of the time required for the total treatment planning is due to unavailability of the target volumes from physicians, and the average amount of time spent by physicians is about 8, 7, and 18 hours for prostate, lung, and head and neck IMRT, respectively [2]. Therefore, the dose prediction with patient contours does not reduce the time of total treatment planning for IMRT as substantially as might be hoped. If the dose distribution could be predicted without the patient contours, it would save much time for treatment planning, allowing physicians and dosimetrists to focus their expertise on more challenging cases or demanding tasks.

The goals of the present study were to develop a fully automated dose generation framework for IMRT of prostate cancer by directing a GAN to learn the patient CT datasets without the contour information, and to compare its prediction performance to a conventional prediction model trained from patient contours. To our knowledge, this is the first report to predict the dose distribution for IMRT using only CT images.

## Materials and methods

**Fig 1:**
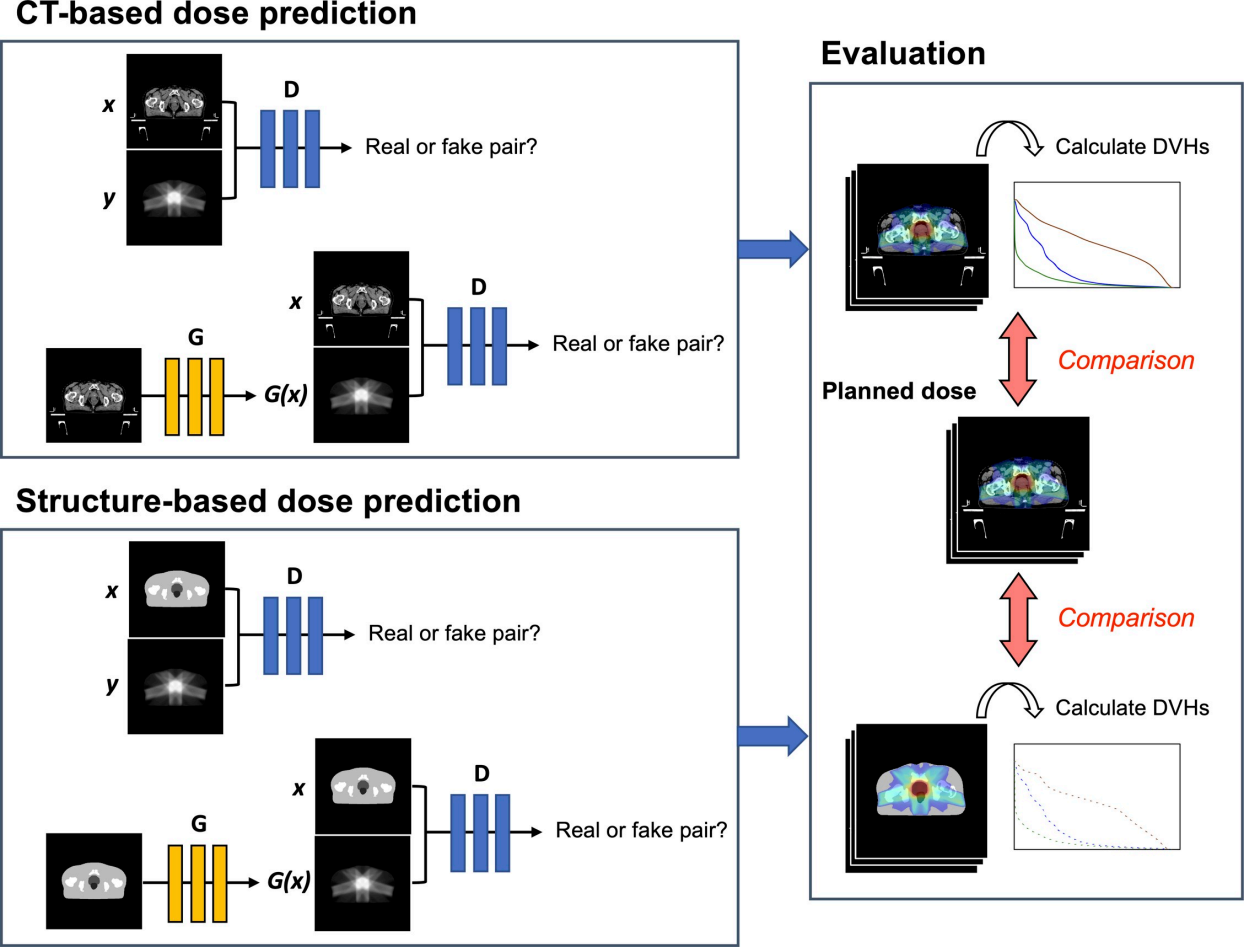
Overall framework of our proposed methods. Two prediction models of dose distribution for IMRT were created using the GAN: a CT-based dose prediction model and a structure-based dose prediction model. Various dosimetric parameters were calculated from the dose-volume-histograms (DVHs) of the generated dose distribution in each model, and the dosimetric parameters in each model were compared to those in the original plans.

The overall framework of our approach is shown in Fig 1. We used a novel framework to predict the dose distribution of IMRT for prostate cancer based on the GAN. This framework did not require the use of contour information or the selection of a range of input CT images; instead, a whole CT dataset was used for the prediction. To evaluate the accuracy of the CT-based prediction, a conventional structure-based prediction model was created, and various dosimetric parameters were compared between the CT-based model and structure-based model.

### Patients

Ninety prostate cancer patients were used for training and testing. All patients had primary prostate cancer and underwent 5-field IMRT between May 2007 and November 2013 at our institution. All patients were prescribed 78 Gy/39 fractions to a planning target volume (PTV) that could be covered with 95% of the prescribed dose (D_95%_ to 78 Gy). The PTV was created by adding a margin of 5 mm in all directions to the clinical target volume (CTV). The CTV was delineated to include the prostate with a margin of 5 mm excluding the rectum, while containing the base of the seminal vesicles. If parts of the rectum and bowel were present in the PTV, a modified PTV excluding these tissues was generated, and 95% of the modified PTV received the prescribed dose. The beam arrangements of 5-field IMRT were the same (255, 315, 45, 105, 180) across all patients, and the median PTV volume was 113.8 cm^3^ (range: 79.3−292.1 cm^3^). The details of the indications for IMRT at our institution have been described previously [13]. The study was approved by the ethics committee of the cancer institute hospital of Japanese foundation for cancer research (2019–1053).

### Pre-processing

To create the pairs of precisely aligned source and target images, the matrix size of RT-dose images was converted to 512 × 512 pixels with 16 bits to match the size of the CT images, because the matrix size of the RT-dose was different in each patient due to the variation in body size. The resolution of RT-doses was set to 1 × 1 mm from 2.5 × 2.5 mm with bilinear interpolation in order to confirm the capability of image-to-image translation using GAN in radiotherapy. The resolution of CT and structure images was also set to 1 × 1 mm and the slice thickness was 2 or 3 mm in all patients. All dose images were saved in units of cGy. The PTV, bladder, rectum, bone and body were used for the input structure images.

### The frameworks for generative adversarial networks

The pix2pix, is one of the supervised learning techniques adopted in GANs, was applied for translating CT or structure images into the dose distribution of IMRT. GANs are widely used for image-to-image translation of medical images such as the tasks for super resolution [14], noise reduction [15], and cross-modality synthesis [16–18]. The idea of pix2pix was proposed by Isola et al. [19] based on a conditional GAN that could synthesize images from pairs of precisely aligned image datasets consisting of source and target images—e.g., the CT and structure images fall into the category of source images, while the corresponding RT-dose images are considered target images in the present study. The GAN is constructed from the generator and discriminator parts. The U-net-based architecture is used for the generator, while a convolutional PatchGAN classifier is used for the discriminator [19]. The generator is trained to produce a simulated dose distribution that cannot be distinguished from the "real" dose distribution images, while the discriminator is trained to detect the generator’s “fakes” as well as possible (Fig 1). The objective of pix2pix $L_{GAN}(G,D)$ can be expressed with the generator *G* and the discriminator *D*:

$$L_{GAN}(G,D)=E_{x,y}[\log\;D(x,y)]+E_{x}[\log\left(1-D(x,G(x)\right)],$$ (1)

where *x* is the source image (i.e., the CT or structure image), *y* is the target image (i.e., the corresponding RT-dose image), *G*(*x*) is the dose image produced by the generator, *D*(*x*,*y*) is the probability that the real pair (*x*,*y*) was correctly discriminated as real by the discriminator and *D*(*x*,*G*(*x*)) is the probability that the fake pair (*x*,*G*(*x*)) was correctly discriminated as fake by the discriminator. The probability is represented as a binary problem, i.e., *D*(*x*,*y*) or *D*(*x*,*G*(*x*)) → [1, 0], where 1 suggests that the discriminator predicts the input-paired images as real and 0 suggests that the discriminator predicts the input-paired images as fake. If the discriminator could completely identify the input images as real or fake, the objective would be increased. In contrast, the generator tries to minimize this objective (i.e., to produce an image that fools the discriminator: *D*(*x*,*G*(*x*)) → 1). The relationships can be expressed by $\underset{G}{min}\;\underset{D}{max}\;L_{GAN}(G,D)$, as in a minimax game. In order to attain the fast convergence and stable training for the network, the generator measures how close the images of the real dose distribution *y* are to the images of the generated dose distribution *G(x)* by using the L1-distance $L_{L1}(G)$:

$$L_{L1}(G)=E_{x,y}[{\left\|y-G(x)\right\|}_{1}].$$ (2)

The final objectives of the pix2pix $\hat{G}$ can be expressed by combining Eqs (1) and (2):

$$\hat{G}=\arg\;\underset{G}{\min}\;\underset{D}{\max}\;L_{GAN}(G,D)+{\lambda L}_{1}(G),$$ (3)

where *λ* is the weight on the L1 term for the generator. The details of the architecture are provided in Isola et al. [19].

### Training and testing

The total 90 patients were divided into groups of 81 patients (90%) for training and 9 patients (10%) for testing. The details of patient characteristics assigned to training or testing were shown in S1 Table. The number of images in each CT, structure and RT-dose was 7467 for the training and 876 for the testing, respectively. We did not select the range of input CT images because the generalizability of the generated dose distribution around the target should be confirmed. In addition, the range of input structure images was not selected for the structure-based model. The prediction models were trained with a GPU (NVIDIA GeForce GTX 1080 Ti). The Adam solver was applied to optimization, with a learning rate of 0.0002, and momentum parameters for the Adam were *β*_1_ = 0.5 and *β*_2_ = 0.999, respectively. The batch size was set to 4. The patch size of 70 × 70 was used for the discriminator receptive fields. The weight on L1 term *λ* was set to 100. The number of trained parameters was 57,190,084 in both prediction models. The training iterations in the CT-based prediction model and the structure-based prediction model were selected as 400k (215 epochs), and 300k (160 epochs), respectively. These were empirically determined based on the preliminary experiments. Finally, the dose distributions of the 9 test cases were predicted by using the corresponding trained model with CT or structures.

### Evaluation

Evaluated dosimetric parameters of PTV and OARs are shown in Table 1. To compare the prediction performance between the CT-based model and the structure-based model, the dose differences of the dosimetric parameter between the original plan (ground truth) and the prediction were calculated as

$$Dose\;difference=\frac{D_{prediction}-D_{grond\;truth}}{D_{prescription}}∙100,$$ (4)

where *D*_*prediction*_ means any objective dosimetric parameter calculated from the predicted dose distribution, *D*_*ground truth*_ represents the corresponding dosimetric parameters calculated from the original dose distribution, and *D*_*prescription*_ is the prescribed dose to PTV. We used the index of Nguyen et al. [4] with slight changes; for our present purposes, the dose difference was calculated by subtracting the planned dose from the predicted dose to confirm whether the generated dose distribution is an overdose or underdose. The dose differences were calculated using only dose-specific parameters such as D_x%_ and D_mean_, while the absolute volume difference calculated by subtracting the ground truth from the prediction was used for evaluating the volume-specific parameters such as V_xGy_. The mean dose differences in all test cases were compared between the CT-based model and the structure-based model. The conformation number (CN) [20] was defined as

$$CN=\frac{{Vt}_{100}^{2}}{\left({Vt}_{vol}∙V_{100}\right)},$$ (5)

where *Vt*_*100*_ is the target volume receiving at least the prescribed dose, *Vt*_*vol*_ is the target volume, and *V*_*100*_ is the total volume receiving at least the prescribed dose. A value close to unity means identical target coverage. The homogeneity index (HI) [21] was defined as

$$HI=\frac{D_{5\%}-D_{95\%}}{D_{mean}},$$ (6)

where *D*_*x%*_ is the dose received by ≥ x% of the PTV volume and *D*_*mean*_ denotes the mean dose to the PTV volume. A value close to zero means identical target homogeneity. The subtracted dose distribution between the ground truth and the prediction was created, and compared between the CT-based model and the structure-based model. The dose profile at the iso-center plane was also compared among the three approaches (ground truth vs. CT-based model vs. structure-based model). Finally, the mean prediction time in all the test cases was calculated for the evaluation.

**Table 1:** Evaluated dosimetric parameters.

Objects	Criteria	Objects	Criteria
PTV	D_98%_ [cGy]	Bladder, Rectum	D_max_ [cGy]
	D_95%_ [cGy]		D_2%_ [cGy]
	D_50%_ [cGy]		D_mean_ [cGy]
	D_2%_ [cGy]		V_50_ [%]
	D_mean_ [cGy]		V_60_ [%]
	CN		V_70_ [%]
	HI	Body	D_max_ [cGy]
			D_mean_ [cGy]
		Femoral head_L, R	D_max_ [cGy]
			D_mean_ [cGy]

D_x%_: dose received by ≥ x% of the objective structure; V_x_: the volume receiving x Gy.

CN: conformation number. HI: homogeneity index.

## Results

### Prediction model

The training time was 131,286 seconds for the CT-based prediction model and 99,065 seconds for the structure-based prediction model. The prediction time per patient (mean ± SD) was 4.93 ± 0.27 seconds. The generator loss, the discriminator loss and the loss for the L1-distance in each prediction model are shown in the Supplemental materials (S1 and S2 Figs).

### Dose distribution

**Fig 2:**
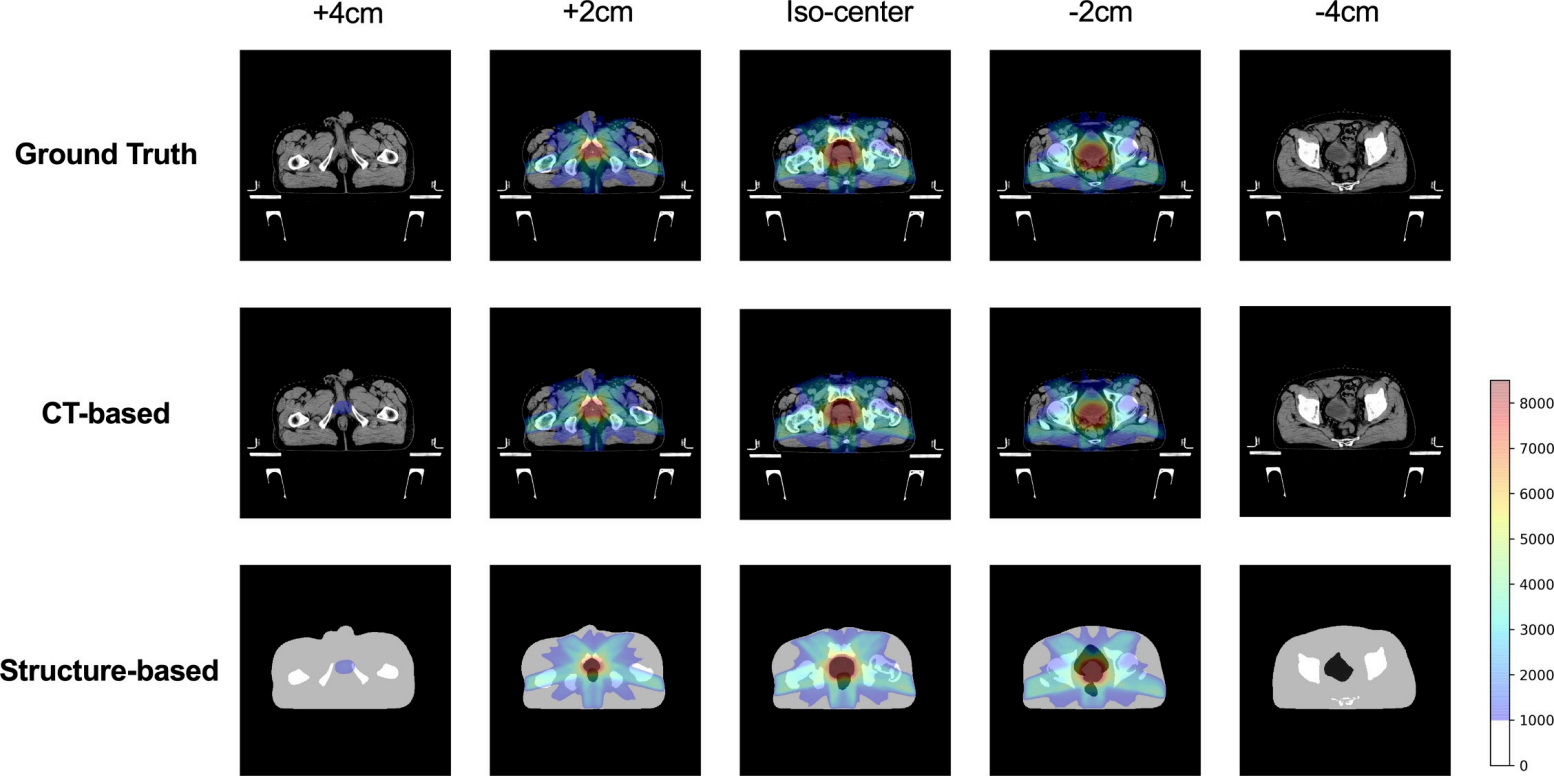
Comparison of dose distribution in the ground truth and both prediction models. The highlighted dose distribution ranges from 1000 cGy to 8500 cGy.

**Fig 3:**
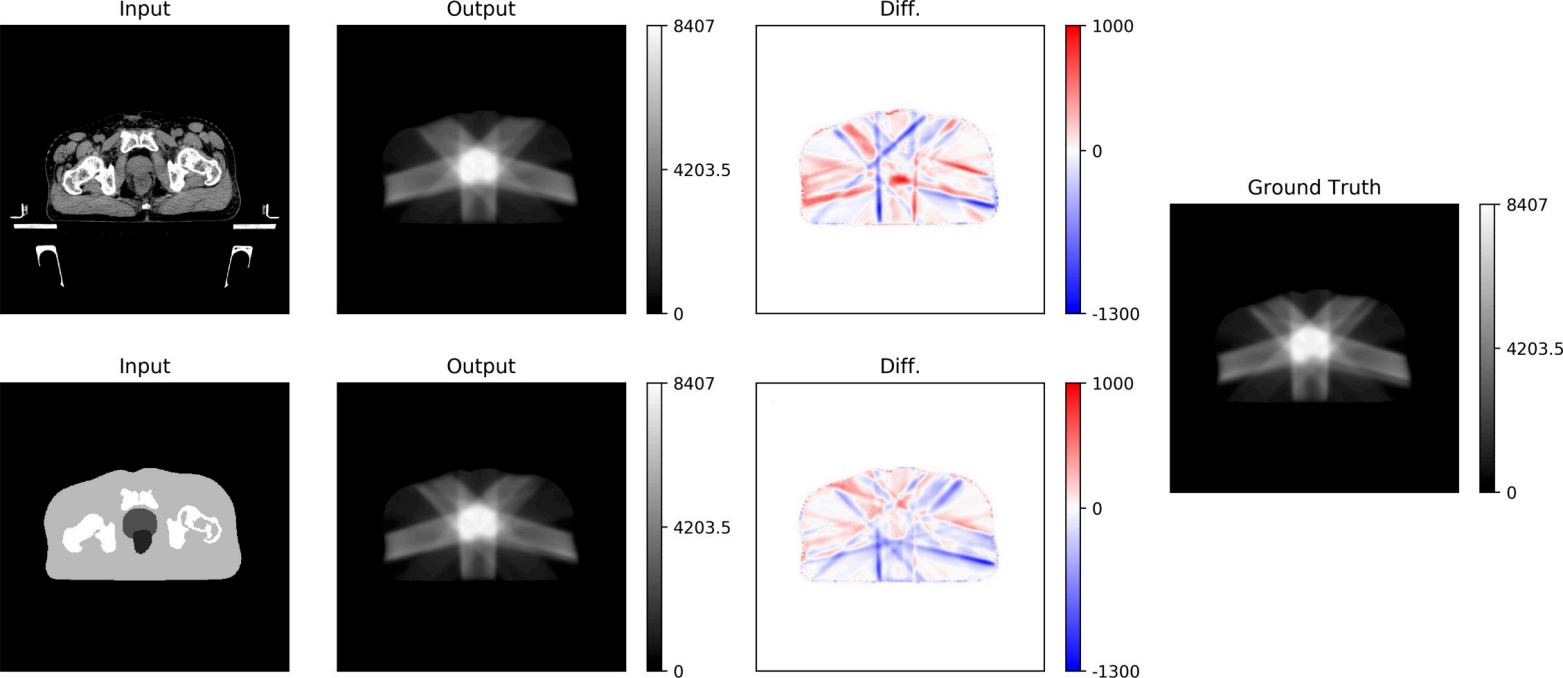
Dose distribution at iso-center, calculated by subtracting the dose distribution in the ground truth from the generated dose distribution in the same patient as shown in Fig 2. The highlighted dose differences range from −1300 cGy to 1000 cGy.

**Fig 4:**
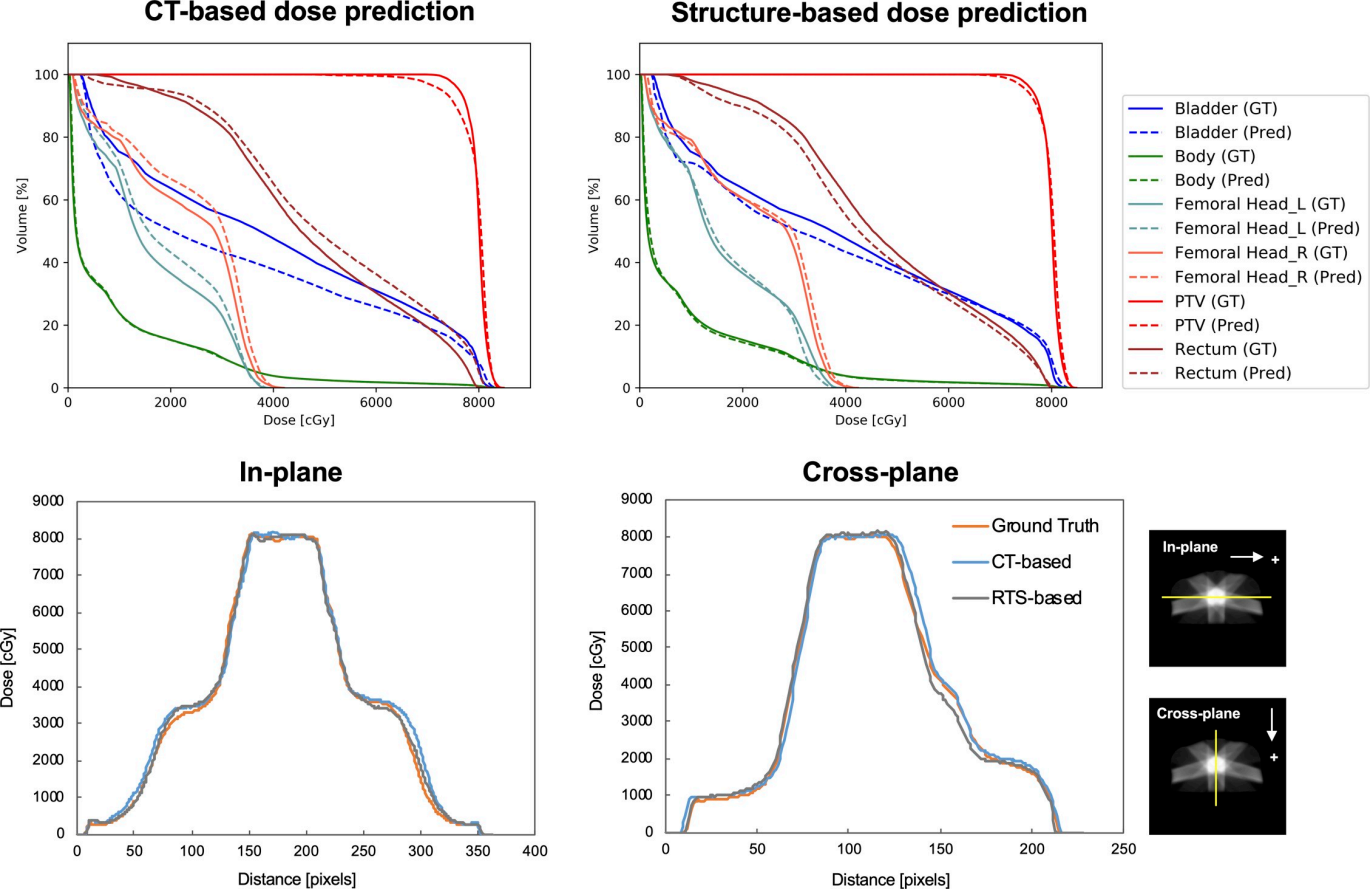
First row: Comparison of DVHs with solid lines (ground truth) and dotted lines (prediction). **Second row: Comparison of dose profiles in the iso-center plane among the three approaches.** The case shown is the same as that depicted in Figs 2 and 3. The direction to the horizontal line on the center of the axial dose distribution was named the in-plane direction and the direction to the vertical line was named the cross-plane direction.

The results of the dose-distribution comparison in one test case are summarized in Fig 2, and the dose distribution derived by subtracting the ground truth from the prediction is shown in Fig 3. The small area irradiated by a low-dose was observed on the slice 4 cm distant from the iso-center in both prediction models, while there were no doses in the area of the −4 cm slice (Fig 2). Notable dose differences were observed along the beam path in both prediction models (Fig 3). Results of the comparison of dose-volume-histograms (DVHs) and dose profiles in the iso-center plane are shown in Fig 4. The dose profiles in both prediction models were in good agreement with those in the ground truth. In particular, the dose profiles of the rectum side in the cross-plane well reflected the DVH curves of the rectum, and dose reduction was found in the structure-based prediction model compared with the ground truth.

### Dosimetric parameters

**Fig 5:**
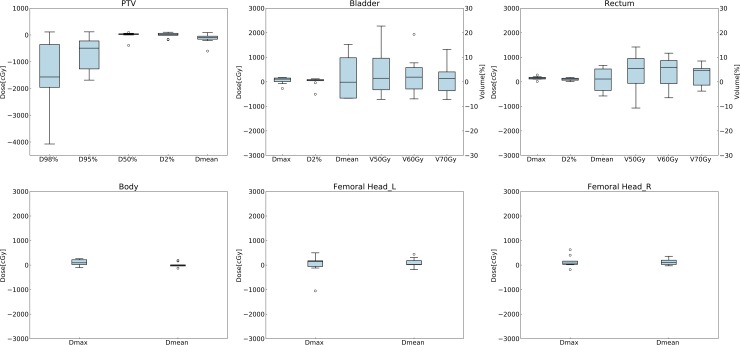
Box plots of the CT-based prediction model showing the absolute differences of dosimetric parameters between the ground truth and the prediction in all test cases. The difference was calculated by subtracting the ground truth from the prediction. The first axis shows the absolute dose differences for dose-specific parameters such as D_x%_, and the second axis shows the absolute volume differences for volume-specific parameters such as V_xGy_.

**Fig 6:**
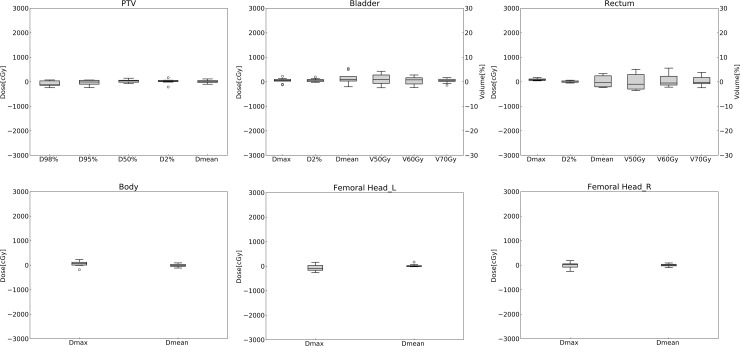
Box plots of the structure-based prediction model showing the absolute differences of dosimetric parameters between the ground truth and the prediction in all test cases. The difference was calculated by subtracting the ground truth from the prediction. The first axis shows the absolute dose differences for dose-specific parameters such as D_x%_, and the second axis shows the absolute volume differences for volume-specific parameters such as V_xGy_.

Table 2 summarizes the average dose differences and absolute volume differences in all test cases for PTV and OARs. The dose differences in the CT-based prediction model were within approximately 2% for PTV except for the parameters D_98%_ and D_95%_ and within approximately 3% for OARs, while the dose differences in the structure-based prediction model were within approximately 1% for PTV and approximately 2% for OARs. The absolute volume differences in the CT-based prediction model and the structure-based prediction model were within approximately 3% and 1% on average, respectively. Table 3 shows the comparison of dose distributions by the CN and the HI. Although the CN and HI in the CT-based prediction model were inferior to those of the ground truth, the CN and HI in the structure-based prediction model were comparable to those of the ground truth. Statistical results of the evaluated dosimetric parameters in PTV and OARs were shown in S2 Table. Box plots showing the absolute differences of dosimetric parameters in all test cases are shown in Figs 5 and 6. Although worse target coverage such as for the parameter D_98%_ was observed in the CT-based prediction model, small dose deviations were seen in the PTV and the OARs through all the test cases in the structure-based prediction model.

**Table 2:** Summary of dose differences and absolute volume differences in PTV and OARs.

Objects	Criteria	CT-based	Structure-based	Objects	Criteria	CT-based	Structure-based
**PTV**	D_98%_	−19.05 ± 0.18	−0.96 ± 0.01	**Rectum**	D_max_	1.91 ± 0.01	1.14 ± 0.01
	D_95%_	−8.67 ± 0.08	−0.40 ± 0.01		D_2%_	1.26 ± 0.01	0.17 ± 0.01
	D_50%_	−0.08 ± 0.02	0.38 ± 0.01		D_mean_	0.66 ± 0.06	−0.02 ± 0.03
	D_2%_	−0.01 ± 0.01	0.21 ± 0.01		V_50_	3.08 ± 8.54	0.18 ± 3.44
	D_mean_	−1.67 ± 0.03	0.20 ± 0.01		V_60_	3.15 ± 6.82	0.60 ± 2.92
**Bladder**	D_max_	0.69 ± 0.02	0.56 ± 0.01		V_70_	2.48 ± 4.59	0.39 ± 2.13
	D_2%_	−0.02 ± 0.03	0.87 ± 0.01	**Body**	D_max_	1.22 ± 0.02	0.76 ± 0.02
	D_mean_	2.39 ± 0.11	2.03 ± 0.03		D_mean_	0.05 ± 0.01	−0.18 ± 0.01
	V_50_	3.19 ± 2.45	1.15 ± 2.45	**Femoral head_L**	D_max_	0.29 ± 0.06	−0.78 ± 0.02
	V_60_	2.42 ± 8.08	0.43 ± 1.62		D_mean_	1.35 ± 0.02	0.28 ± 0.01
	V_70_	1.25 ± 6.23	0.39 ± 0.96	**Femoral head_R**	D_max_	1.89 ± 0.03	−0.33 ± 0.02
					D_mean_	1.68 ± 0.02	0.01 ± 0.01

Value = mean ± SD, Dose differences [%] = [(***D***_***prediction***_−***D***_***ground truth***_)/***D***_***prescription***_]∙**100**, Absolute volume differences [%] = ***V***_***prediction***_−***V***_***ground truth***_

**Table 3:** Comparison of dose distribution by CN and HI.

Criteria	Ground Truth	CT-based	Structure-based
CN	0.84 ± 0.02	0.67 ± 0.14	0.83 ± 0.04
HI	0.08 ± 0.02	0.17 ± 0.09	0.09 ± 0.01

CN: conformation number; HI: homogeneity index.

## Discussion

The present study developed the fully automated dose generation framework for IMRT of prostate cancer by allowing a GAN to learn the features of CT images and compared its prediction performance to the conventional prediction framework trained from the contour information. Although the prediction performance of the structure-based model was superior to that of the CT-based prediction model, we demonstrated that the dose differences of all dosimetric parameters except for D_98%_ and D_95%_ for PTV were within approximately 2% and approximately 3% for OARs in the CT-based dose prediction model, with a mean prediction time of approximately 5 seconds per patient. Table 4 compares the prediction performance with the average absolute dose differences evaluated by Nguyen et al. [4]. The average absolute dose differences of most evaluation metrics in the structure-based model were smaller than those of Nguyen et al., and the prediction performance was comparable to the previous results even when the CT images were used for the training. These results suggested that the iterative optimization process for the IMRT planning could be reduced by using the CT-based prediction model, because the treatment planner can know which areas should receive increased doses or which areas should receive decreased doses based on the results of the prediction. Since the CT-based model does not require contour information for the prediction, the time required for the patient contours could be reduced. Moreover, the rapid dose prediction based on the CT images would help to avoid the treatment delay due to the manpower constraints.

**Table 4:** Comparison of prediction performance using the average absolute dose difference*.

Objects	CT-based		Structure-based		Nguyen et al. [4]	
	D_max_	D_mean_	D_max_	D_mean_	D_max_	D_mean_
**PTV**	1.68 ± 0.01	1.98 ± 0.02	1.31 ± 0.01	0.64 ± 0.01	1.80 ± 1.09	1.03 ± 0.62
**Bladder**	1.67 ± 0.01	9.14 ± 0.06	1.21 ± 0.01	2.61 ± 0.02	1.94 ± 1.31	4.22 ± 3.63
**Rectum**	1.91 ± 0.01	5.39 ± 0.02	1.14 ± 0.01	2.34 ± 0.01	1.26 ± 0.62	1.62 ± 1.07
**Body**	1.68 ± 0.01	0.96 ± 0.01	1.31 ± 0.01	0.67 ± 0.01	1.80 ± 1.09	0.48 ± 0.35
**Femoral head_L**	3.92 ± 0.04	1.93 ± 0.02	1.53 ± 0.01	0.45 ± 0.01	3.87 ± 3.26	1.79 ± 1.58
**Femoral head_R**	2.41 ± 0.03	1.77 ± 0.01	1.34 ± 0.01	0.54 ± 0.01	5.07 ± 4.99	2.55 ± 2.38

Value = mean ± SD

*Average absolute dose difference [%] = |(*D*_*ground truth*_−*D*_*prediction*_)/*D*_*prescription*_|∙100

This study is the first attempt to predict the dose distribution for IMRT using only CT images. As a preliminary study, we focused our initial study on prostate cancer patients for confirming the generalizability of the predicted dose distribution by using a GAN framework because the variations of the targets in the prostate cancer patients are relatively small. As a result, this study demonstrated that the dose differences of almost all dosimetric parameters for PTV were within approximately 2% of the prescription dose and approximately 3% for OARs in the CT-based dose prediction model. Therefore, we consider that the CT-based dose prediction could not only help beginners of the IMRT treatment planning for prostate cancer patients to learn which areas should receive increased or decreased doses, but also help some experts to find optimal ways for better treatment planning within a short time in other clinical sites.

Automated treatment planning solutions are widely used for reducing inter-planner variability, reducing the planning time allocated for the optimization process and improving plan quality [22–26]. The important difference between the automated-planning solutions and our proposed method is whether there is an optimization process in treatment planning or not. Since CT-based prediction model is not necessary for the optimization process, physicians or dosimetrists can get achievable dose distribution immediately after CT simulation. The rapid dose prediction based on the CT images might be useful for optimizing a treatment strategy before treatment when radiation therapy can be difficult due to complicated organs placement and re-irradiation, other than the advantage of reducing the total treatment planning time. On the other hand, the plan quality of the RapidPlan, is one of the commercial knowledge-based planning solutions developed by Varian Medical Systems (Palo Alto, CA), was altered depending on the registered model [27], and manual touch-up or additional manual objective was necessary to get equally good IMRT plans [28, 29]. Compared with such knowledge-based planning approach, it is simplified to create the dose prediction model because only the pairs of CT and RT-dose images are needed for the creation of the model.

Although we did not select the range of input CT images for confirming the generalizability of the generated dose distribution around the target, large differences were not seen outside the irradiation fields and good agreements of DVH curves were found (Figs 2–4). However, worse target coverages, such as D_98%_ or the D_95%_, were observed in the CT-based prediction model (Tables 2 and 3). This was caused by an underdose to the PTV margin. The area was covered by a total of the 1 cm margin from the prostate, and therefore GAN could not train the dose distribution for the peripheral area even if the entire CT dataset was trained into the 2D GAN. This is because there was no obvious anatomical structure which showed the concept of the margin around the prostate. For example, the shape of the bladder differs depending on the amount of urine in each patient. This shape variation might have affected the results of training in the doses to the PTV margin on the bladder side. If the entire 3D CT image was trained into the 3D GAN, the underdose to the PTV margin could be fixed. Actually, Babier et al. reported that, compared to the 2D GAN, the 3D GAN better learned the vertical relationship between adjacent axial slices for predicting the dose distribution in oropharyngeal cancer cases [6]. Moreover, although the dose differences were mainly observed along the beam path in both prediction models (Fig 3), we found that the dose differences of the left or right femoral head in the CT-based prediction model and the structure-based prediction model were within approximately 2% and 1% on average, respectively. According to a previous report, the dose differences that were observed along the beam path can be fixed by incorporating both anatomical and beam geometry information into the network [9].

Nguyen et al. predicted the dose for IMRT of prostate cancer patients from patient image contours of PTV and OARs using U-net based architecture, and reported that the average value of the absolute differences in D_max_ and D_mean_ was under 5% of the prescription dose in PTV and OARs [4]. When we compared our results with these previous results, we found that the average absolute dose differences of most evaluation metrics in the structure-based model were smaller than those of Nguyen et al. by using the GAN (Table 4). Some papers demonstrated that the prediction of the GAN outperformed the U-net based architecture [5,11], and this tendency was also seen in the present study (Table 4). Moreover, an extremely small deviation was observed in both prediction models and the prediction performance was comparable to the previous results even when the CT images were used for the training (Table 4). We assumed that the adversarial training between the generator and the discriminator might have contributed to the improvement of prediction performance, because the poor quality outputs from the generator were regarded as "fakes" by the discriminator.

In the pix2pix, PatchGAN was used for the discriminator that only penalizes the structure in local image patches [19], which means the discriminators only look at small patches in an image and try to determine whether each is real or fake. Therefore, even if the part of the rectum was covered from PTV, the generated dose to rectum could be a good prediction of the original dose distribution (Figs 3 and 4). According to a previous report, the use of 70 × 70 receptive fields yields better results for the image-to-image translation task [19], and thus a patch size of 70 × 70 was used for the discriminator receptive fields in the present study. The selection of receptive fields is important to preserve the structure and convert the voxel values. Kida et al. reported that when too large a receptive field was used for the discriminator, the training was affected by the structure and location of the organs, while when the receptive field was too small, the local structural pattern could not be detected and only the voxel values were converted, ignoring the structure [18].

Several limitations in this study bear mention. First of all, although we randomly divided the candidate 90 patients into groups of 81 patients (90%) for training and 9 patients (10%) for testing, the testing cases might not be sufficient to fully evaluate the proposed models. Moreover, the bias of PTV information, such as the target size or the volume in patients assigned to the training cases, might influence the results of predicted dose distribution in the testing cases. However, since there is no significant difference between the training and the testing cases regarding the PTV volume (S3 Fig), the authors consider that the trained cases are extensive. Future work will be focused on increasing the number of testing cases. Second, our prediction model was trained to generate the dose distribution for fixed IMRT planning, rather than the VMAT. Therefore, it is unclear whether the GAN can synthesize an accurate dose distribution for VMAT. However, we consider that it would be easier to train the dose distribution of VMAT than the dose distribution of the fixed IMRT because the VMAT plan has a more continuous dose distribution owing to the rotational irradiation, and the dose differences were mainly observed along the beam path when predicting the dose distribution for IMRT (Fig 3). Finally, when predicting the dose distribution of SIB, it is difficult to train the dose distribution from only the CT datasets, because the anatomical features cannot reflect the dose levels that were determined by the treatment planner. However, the structure-based model could predict the dose distribution of SIB, because the model can learn the dose information determined by the treatment planner from the delineated structure. In fact, some previous works have succeeded in predicting the dose distribution of SIB in head and neck cancer patients by entering the patient structures based on the dose levels into the network [5–8]. However, we hypothesize that the dose distribution of SIB could be generated even with the CT-based prediction model by integrating radiomics analysis into the model. Previous works demonstrated that a radiomics-driven framework can automatically detect the region of prostate cancer or high-risk volumes based on the Gleason score without a human intervention [30–32].

## Conclusions

Accurate and rapid dose prediction was achieved by entering patient CT datasets into the GAN-based framework. The dose differences of all dosimetric parameters except for D_98%_ and D_95%_ for PTV were within approximately 2% and approximately 3% for OARs in the CT-based dose prediction model, while the dose differences of all dosimetric parameters in the structure-based prediction model were within approximately 1% for PTV and approximately 2% for OARs, with a mean prediction time of approximately 5 seconds for IMRT of prostate cancer patients. The rapid dose prediction based on the CT images could reduce the time required for both the iterative optimization process for IMRT and the structure contouring. Thus the total treatment planning time could be greatly shortened, allowing for physicians and dosimetrists to focus their expertise on more challenging cases.

## Supporting information

S1 Fig Average training losses in the generator and discriminator in the CT-based prediction model. (TIFF)

S2 Fig Average training losses in the generator and discriminator in the structure-based prediction model. (TIFF)

S3 Fig Box plots showing the PTV volume in the training or testing patients. There is no significant difference between the training and testing cases. The *P* value was calculated by a welch t-test, with a level of significance set at 5%.
(TIFF)

S1 Table Details of patient characteristics assigned training or testing. (DOCX)

S2 Table Statistical results of the evaluated dosimetric parameters in PTV and OARs. (DOCX)

S3 Table Results of absolute dose or volume differences in all testing cases in the CT-based prediction model. (DOCX)

S4 Table Results of absolute dose or volume differences in all testing cases in the structure-based prediction model. (DOCX)

S5 Table Results of CN and HI calculated from the dose distributions of the CT-based prediction model, the structure-based prediction model and the ground truth. (DOCX)
