# Supplementary figures and images for: Fully automated dose prediction using generative adversarial networks in prostate cancer patients
Source: PLoS One. 2020 May 4;15(5):e0232697. doi: 10.1371/journal.pone.0232697 (PMC7197852; doi:10.1371/journal.pone.0232697)

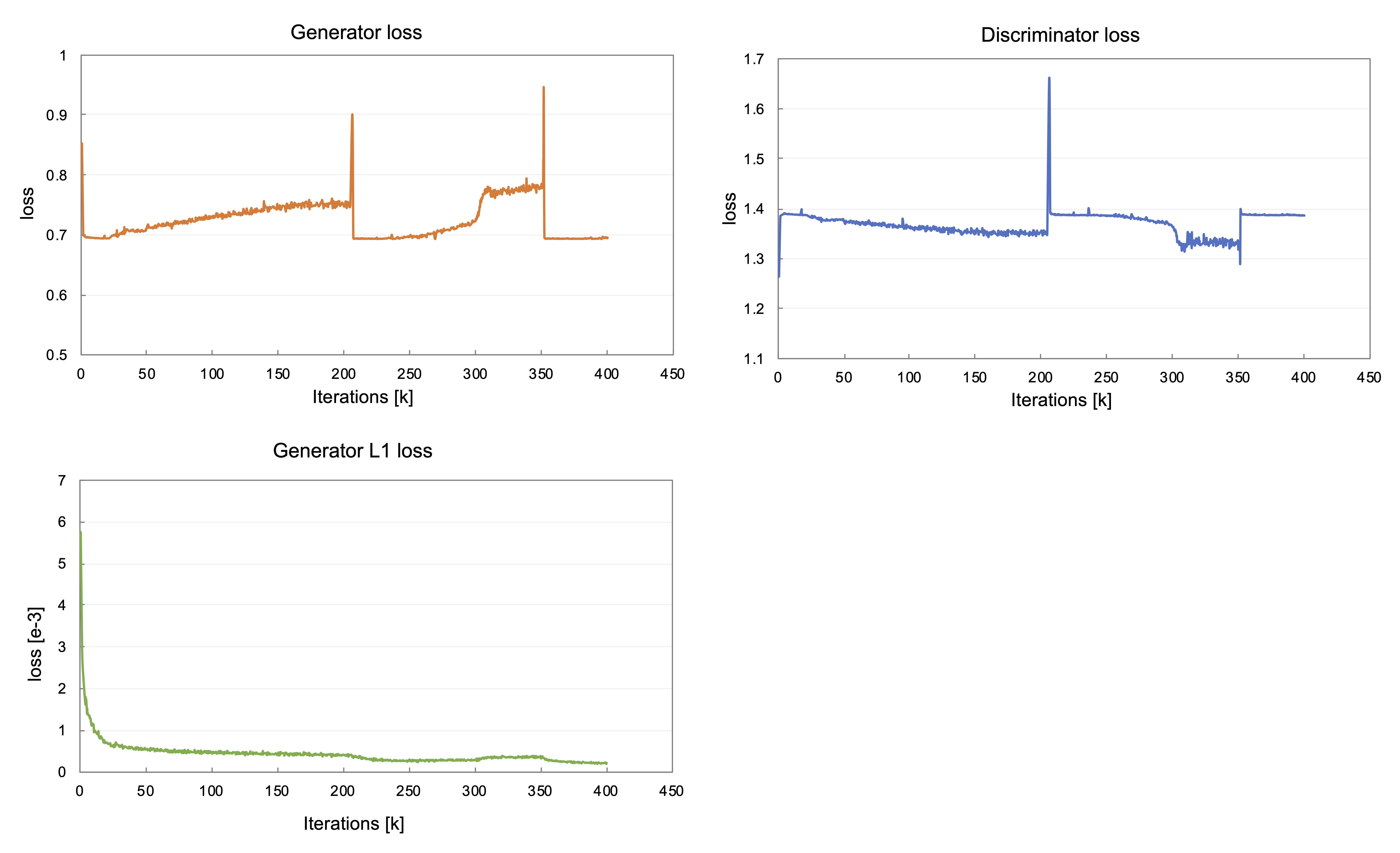

Supplement: S1 Fig — (TIFF) [file pone.0232697.s001.tiff]

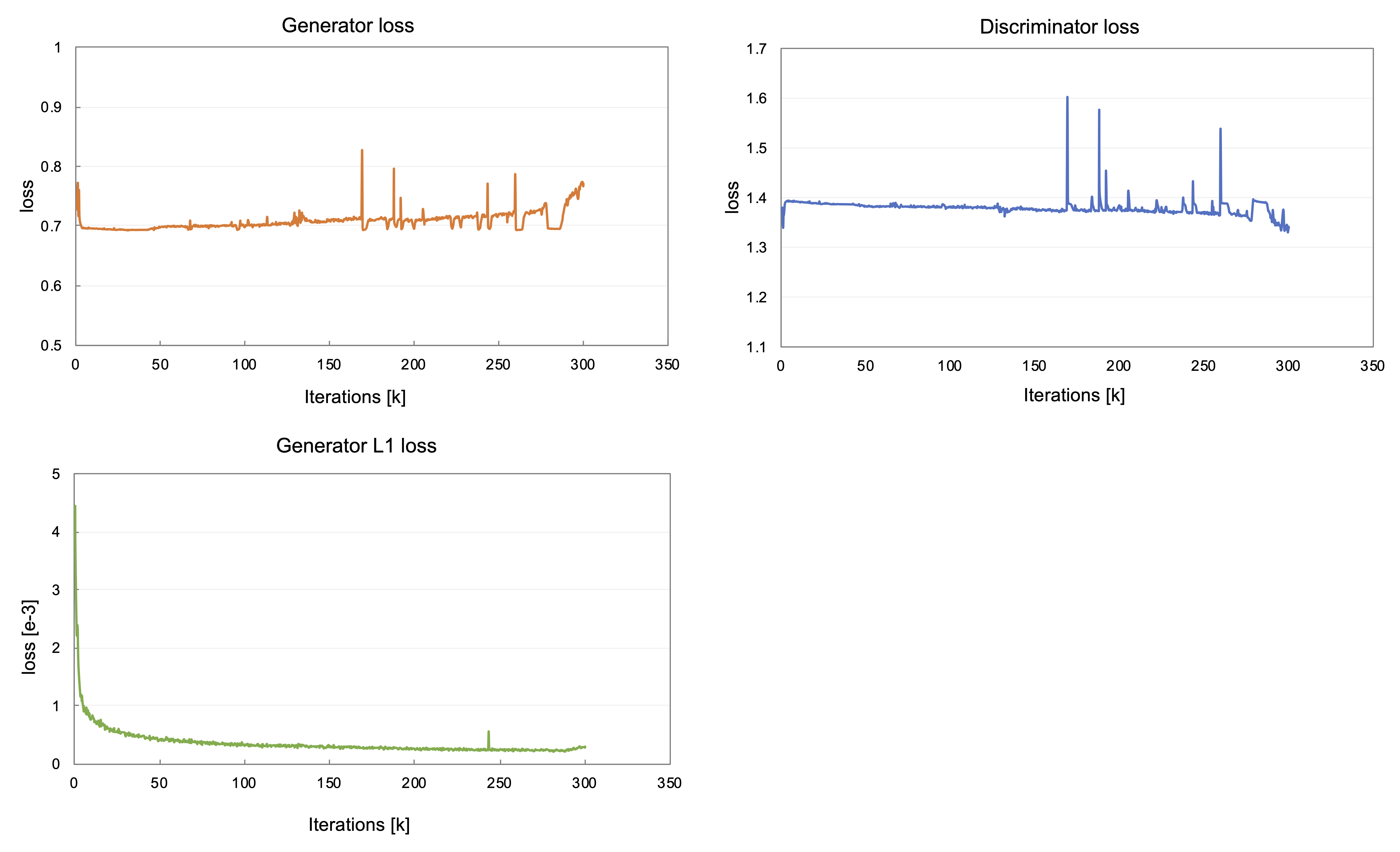

Supplement: S2 Fig — (TIFF) [file pone.0232697.s002.tiff]

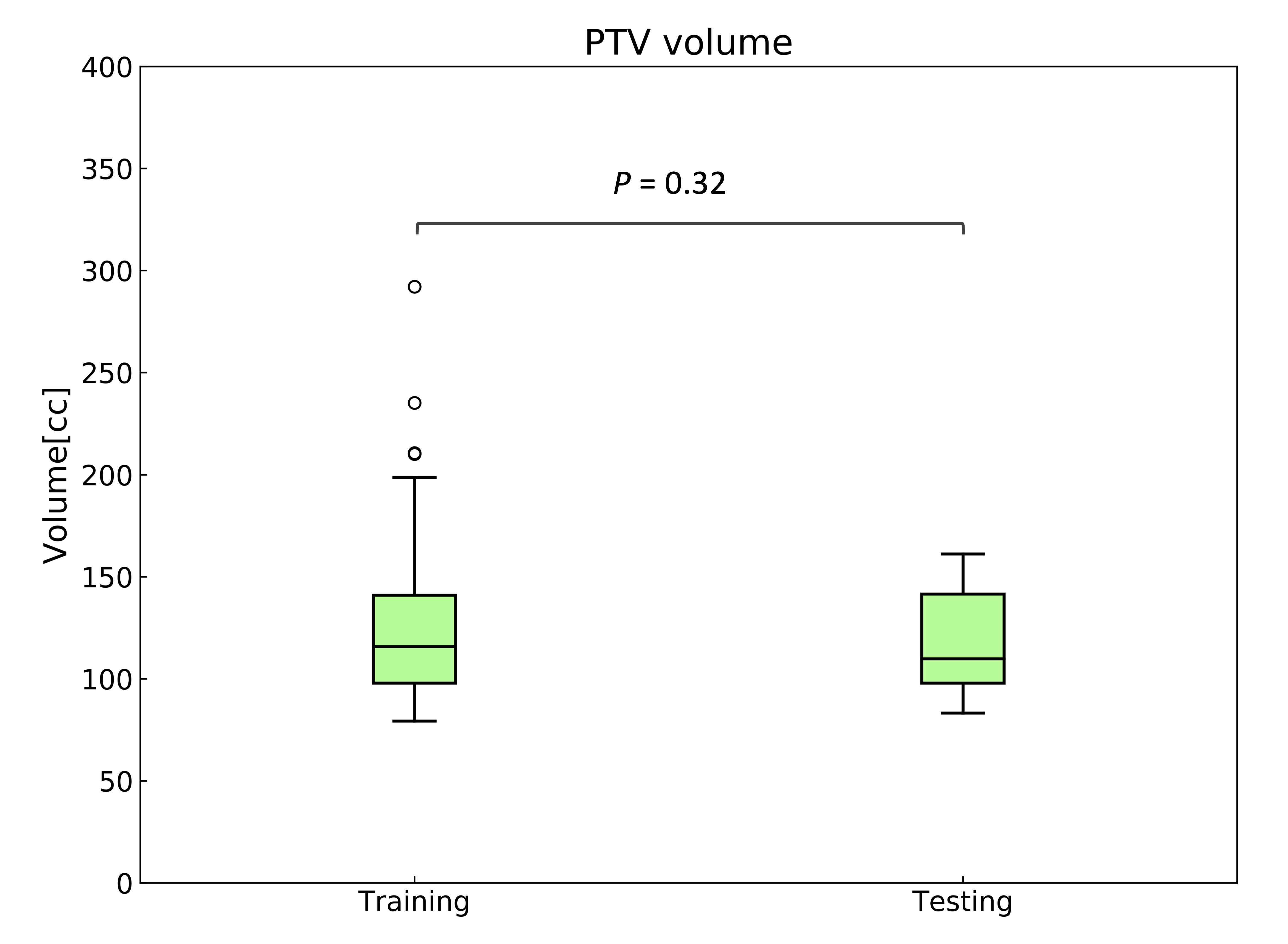

Supplement: S3 Fig — There is no significant difference between the training and testing cases. The P value was calculated by a welch t-test, with a level of significance set at 5%. (TIFF) [file pone.0232697.s003.tiff]
