# Supplementary material for: Fully automated dose prediction using generative adversarial networks in prostate cancer patients
Source: PLoS One. 2020 May 4;15(5):e0232697. doi: 10.1371/journal.pone.0232697 (PMC7197852; doi:10.1371/journal.pone.0232697)
Supplement: S1 Table — (DOCX) [file pone.0232697.s004.docx]

**S1 Table. Details of patient characteristics assigned to training or testing.**

| **Characteristics** | **Training** | **Testing** |
| --- | --- | --- |
| Number of patients | 81 | 9 |
| Age (median) | 55 ~ 82 (69) | 64 ~ 80 (72) |
| iPSA [ng/ml] |  |  |
| iPSA $<$ 10 | 30 | 1 |
| 10 $\leq$ iPSA $\leq$ 20 | 32 | 5 |
| iPSA $>$ 20 | 19 | 3 |
| Grade group (1 / 2 / 3 / 4 / 5) | 5 / 18 / 19 / 19 / 20 | 0 / 3 / 2 / 1 / 3 |
| PTV volume [cc] (median) | 79.3 ~ 292.1 (115.8) | 77.5 ~ 151.8 (103.3) |

iPSA: initial prostate specific antigen.
