# Supplementary material for: Fully automated dose prediction using generative adversarial networks in prostate cancer patients
Source: PLoS One. 2020 May 4;15(5):e0232697. doi: 10.1371/journal.pone.0232697 (PMC7197852; doi:10.1371/journal.pone.0232697)
Supplement: S2 Table — (DOCX) [file pone.0232697.s005.docx]

| **Objects** | **Criteria** | **CT** | **ST** | **GT** | ***P*-value**  **(CT vs. GT)** | ***P*-value**  **(ST vs. GT)** | **Objects** | **Criteria** | **CT** | **ST** | **GT** | ***P*-value**  **(CT vs. GT)** | ***P*-value**  **(ST vs. GT)** |
| --- | --- | --- | --- | --- | --- | --- | --- | --- | --- | --- | --- | --- | --- |
| **PTV** | D_98%_ | 5937.3 | 7348.0 | 7423.0 | 0.01 | 0.11 | **Rectum** | D_max_ | 8237.8 | 8177.6 | 8088.8 | 0.001 | 0.01 |
|  | D_95%_ | 6943.3 | 7588.7 | 7619.5 | 0.01 | 0.38 |  | D_2%_ | 8005.2 | 7919.9 | 7906.8 | 0.01 | 0.56 |
|  | D_50%_ | 8029.6 | 8066.0 | 8036.2 | 0.91 | 0.27 |  | D_mean_ | 4804.6 | 4751.7 | 4753.0 | 0.74 | 0.99 |
|  | D_2%_ | 8368.4 | 8385.4 | 8368.8 | 0.99 | 0.73 |  | V_50_ | 46.7 | 43.8 | 43.6 | 0.32 | 0.94 |
|  | D_mean_ | 7883.8 | 8029.9 | 8014.2 | 0.09 | 0.56 |  | V_60_ | 33.3 | 30.7 | 30.1 | 0.20 | 0.75 |
|  | CN | 0.67 | 0.83 | 0.84 | 0.01 | 0.31 |  | V_70_ | 20.2 | 18.1 | 17.7 | 0.15 | 0.73 |
|  | HI | 0.17 | 0.09 | 0.08 | 0.01 | 0.29 | **Body** | D_max_ | 8595.8 | 8559.7 | 8500.6 | 0.14 | 0.28 |
| **Bladder** | D_max_ | 8237.8 | 8177.6 | 8088.8 | 0.43 | 0.42 |  | D_mean_ | 860.2 | 841.8 | 856.2 | 0.92 | 0.71 |
|  | D_2%_ | 8005.2 | 7919.9 | 7906.8 | 0.08 | 0.13 | **FH_L** | D_max_ | 4148.2 | 4064.0 | 4125.2 | 0.88 | 0.62 |
|  | D_mean_ | 4804.6 | 4751.7 | 4753.0 | 0.77 | 0.80 |  | D_mean_ | 1754.0 | 1670.4 | 1648.3 | 0.48 | 0.85 |
|  | V_50_ | 46.7 | 43.8 | 43.6 | 0.66 | 0.87 | **FH_R** | D_max_ | 4167.4 | 3994.4 | 4020.0 | 0.35 | 0.85 |
|  | V_60_ | 33.3 | 30.7 | 30.1 | 0.68 | 0.94 |  | D_mean_ | 1740.6 | 1610.4 | 1609.4 | 0.41 | 0.99 |
|  | V_70_ | 20.2 | 18.1 | 17.7 | 0.78 | 0.93 |  |  |  |  |  |  |  |

**S2 Table. Statistical results of the evaluated dosimetric parameters in PTV and OARs.**

Value = mean, CT: CT-based prediction model; ST: structure-based prediction model; GT: ground truth; FH_L: left femoral head; FH_R: right femoral head; CN: conformation number; HI: homogeneity index.
