# Supplementary material for: Fully automated dose prediction using generative adversarial networks in prostate cancer patients
Source: PLoS One. 2020 May 4;15(5):e0232697. doi: 10.1371/journal.pone.0232697 (PMC7197852; doi:10.1371/journal.pone.0232697)
Supplement: S3 Table — (DOCX) [file pone.0232697.s006.docx]

**S3 Table. Results of absolute dose or volume differences in all testing cases in the CT-based prediction model.**

| Objects | Metric | **Testing patients** | | | | | | | | |
| --- | --- | --- | --- | --- | --- | --- | --- | --- | --- | --- |
|  |  | PT1 | PT2 | PT3 | PT4 | PT5 | PT6 | PT7 | PT8 | PT9 |
| **PTV** | D_98%_ | -359.5 | -4075.1 | -249.4 | 112.3 | -3011.0 | -1955.7 | -1570.4 | -1694.9 | -566.8 |
|  | D_95%_ | -224.2 | -492.2 | -65.8 | 116.4 | -1506.5 | -1686.7 | -1267.6 | -618.8 | -340.9 |
|  | D_50%_ | 0.8 | 63.9 | 38.7 | 93.8 | 43.4 | -388.5 | 37.4 | 17.6 | 33.8 |
|  | D_2%_ | -167.3 | 63.5 | 26.0 | 103.2 | 91.8 | -179.3 | 23.4 | 45.9 | -10.6 |
|  | D_mean_ | -50.1 | -93.2 | 21.9 | 87.0 | -211.8 | -601.3 | -157.9 | -123.9 | -44.1 |
| **Bladder** | D_max_ | -73.0 | 87.0 | 156.0 | 170.0 | 153.0 | -271.0 | 11.0 | 143.0 | 110.0 |
|  | D_2%_ | 43.3 | -41.1 | 44.7 | 120.2 | 80.3 | -507.1 | 52.0 | 97.0 | 93.4 |
|  | D_mean_ | 1193.7 | -664.2 | 979.8 | 1527.1 | -668.7 | -356.5 | 345.7 | -14.5 | -664.6 |
|  | V_50_ | 10.6 | -2.9 | 9.6 | 22.7 | -7.1 | -3.2 | 4.7 | 1.5 | -7.2 |
|  | V_60_ | 5.8 | -1.9 | 7.7 | 19.3 | -7.0 | -2.9 | 4.0 | 1.9 | -5.2 |
|  | V_70_ | 4.0 | -1.9 | 6.1 | 13.2 | -7.3 | -3.6 | 3.1 | 1.4 | -3.9 |
| **Rectum** | D_max_ | 151.0 | 205.0 | 132.0 | 179.0 | 276.0 | 14.0 | 109.0 | 115.0 | 160.0 |
|  | D_2%_ | 113.3 | 175.9 | 66.3 | 145.1 | 111.5 | 76.6 | 11.4 | 10.4 | 175.4 |
|  | D_mean_ | 551.5 | 114.2 | 521.9 | 667.7 | -351.0 | -577.0 | -491.7 | -239.2 | 268.2 |
|  | V_50_ | 7.4 | 9.5 | 10.8 | 14.2 | -0.2 | -10.7 | -7.9 | -0.6 | 5.4 |
|  | V_60_ | 5.9 | 9.7 | 8.7 | 11.7 | -0.7 | -6.5 | -6.4 | 0.1 | 6.0 |
|  | V_70_ | 4.7 | 6.9 | 5.2 | 8.5 | -1.3 | -3.1 | -3.8 | -0.2 | 5.4 |
| **Body** | D_max_ | -104.0 | 228.0 | 81.0 | 216.0 | 258.0 | -56.0 | 91.0 | 120.0 | 23.0 |
|  | D_mean_ | -37.0 | -15.1 | 182.5 | 164.6 | -126.1 | -128.2 | -11.5 | -1.3 | 8.1 |
| **FH_L** | D_max_ | 488.0 | 172.0 | 169.0 | -44.0 | 498.0 | -1053.0 | -55.0 | 152.0 | -120.0 |
|  | D_mean_ | 20.8 | 14.7 | 441.3 | 22.7 | -14.5 | -187.0 | 306.5 | 167.0 | 179.4 |
| **FH_R** | D_max_ | 625.0 | 81.0 | 403.0 | 144.0 | 29.0 | -184.0 | 53.0 | 161.0 | 15.0 |
|  | D_mean_ | 109.5 | 24.6 | 357.2 | -31.4 | 5.0 | 155.4 | 107.4 | 253.8 | 198.9 |

PT: patient; FH_L: left femoral head; FH_R: right femoral head.

Absolute dose differences [cGy] = $D_{prediction}-D_{ground truth}$, Absolute volume differences [%] = $V_{prediction}-V_{ground truth}$
