# Supplementary material for: Fully automated dose prediction using generative adversarial networks in prostate cancer patients
Source: PLoS One. 2020 May 4;15(5):e0232697. doi: 10.1371/journal.pone.0232697 (PMC7197852; doi:10.1371/journal.pone.0232697)
Supplement: S4 Table — (DOCX) [file pone.0232697.s007.docx]

**S4 Table. Results of absolute dose or volume differences in all testing cases in the structure-based prediction model.**

| Objects | Metric | **Testing patients** | | | | | | | | |
| --- | --- | --- | --- | --- | --- | --- | --- | --- | --- | --- |
|  |  | PT1 | PT2 | PT3 | PT4 | PT5 | PT6 | PT7 | PT8 | PT9 |
| **PTV** | D_98%_ | 65.5 | 35.5 | 74.1 | -143.3 | -22.5 | -242.2 | -126.0 | -132.7 | -182.6 |
|  | D_95%_ | 60.1 | 65.9 | 72.4 | -9.3 | 21.0 | -239.3 | -45.1 | -97.5 | -105.8 |
|  | D_50%_ | -34.7 | 81.4 | 47.9 | 145.8 | 16.2 | -68.8 | 61.1 | -15.6 | 35.6 |
|  | D_2%_ | -214.1 | 64.2 | 57.4 | 164.3 | 33.5 | -13.4 | 11.1 | 26.9 | 19.5 |
|  | D_mean_ | -31.3 | 71.2 | 48.2 | 114.6 | 19.0 | -99.2 | 32.1 | -22.9 | 9.7 |
| **Bladder** | D_max_ | -128.0 | 26.0 | 62.0 | 134.0 | 222.0 | -100.0 | 31.0 | 97.0 | 49.0 |
|  | D_2%_ | 12.8 | 64.9 | 11.3 | 128.7 | 194.8 | -21.4 | 67.4 | 64.0 | 85.4 |
|  | D_mean_ | 494.4 | 214.1 | 543.9 | 85.1 | 20.2 | 30.4 | 84.8 | 155.7 | -203.8 |
|  | V_50_ | 4.2 | 0.8 | 2.8 | -2.5 | 2.3 | -0.7 | 0.9 | 4.3 | -1.8 |
|  | V_60_ | 1.7 | 0.4 | 1.6 | -2.4 | 1.0 | -0.9 | 0.8 | 2.8 | -1.1 |
|  | V_70_ | 1.0 | 0.1 | 0.6 | -1.4 | 1.3 | -0.7 | 0.6 | 1.7 | 0.3 |
| **Rectum** | D_max_ | 56.0 | 114.0 | 66.0 | 173.0 | 78.0 | 85.0 | 143.0 | 38.0 | 46.0 |
|  | D_2%_ | 20.5 | 32.9 | 45.1 | 65.1 | -28.1 | -20.3 | 29.0 | -50.6 | 24.8 |
|  | D_mean_ | 329.9 | 241.0 | 245.1 | -15.5 | -38.2 | -230.7 | -112.9 | -206.1 | -224.5 |
|  | V_50_ | 2.9 | 5.1 | 4.9 | -1.2 | 1.0 | -3.6 | -1.0 | -3.0 | -3.5 |
|  | V_60_ | 2.2 | 4.7 | 5.6 | -0.5 | -2.2 | -1.3 | 0.4 | -1.3 | -2.2 |
|  | V_70_ | 1.7 | 2.6 | 3.8 | -0.4 | -2.4 | -0.8 | 1.5 | -0.5 | -2.1 |
| **Body** | D_max_ | -187.0 | 197.0 | 118.0 | 230.0 | 101.0 | 4.0 | 15.0 | 62.0 | -8.0 |
|  | D_mean_ | -117.2 | 33.2 | 92.7 | 43.3 | -112.1 | -49.2 | -12.0 | -1.2 | -7.3 |
| **FH_L** | D_max_ | 29.0 | 10.0 | 159.0 | -103.0 | 65.0 | -266.0 | -187.0 | -165.0 | -93.0 |
|  | D_mean_ | -18.3 | 17.8 | 165.3 | -13.0 | -24.1 | -3.5 | 63.8 | -0.5 | 11.4 |
| **FH_R** | D_max_ | 28.0 | 185.0 | 67.0 | 23.0 | -237.0 | -19.0 | -252.0 | -78.0 | 53.0 |
|  | D_mean_ | 2.8 | 4.8 | 89.9 | 15.0 | -100.9 | -27.8 | -57.9 | 36.1 | 47.3 |

PT: patient; FH_L: left femoral head; FH_R: right femoral head.

Absolute dose differences [cGy] = $D_{prediction}-D_{ground truth}$, Absolute volume differences [%] = $V_{prediction}-V_{ground truth}$
