# Supplementary material for: Fully automated dose prediction using generative adversarial networks in prostate cancer patients
Source: PLoS One. 2020 May 4;15(5):e0232697. doi: 10.1371/journal.pone.0232697 (PMC7197852; doi:10.1371/journal.pone.0232697)
Supplement: S5 Table — (DOCX) [file pone.0232697.s008.docx]

**S5 Table. Results of CN and HI calculated from the dose distributions of the CT-based prediction model, the structure-based prediction model and the ground truth.**

| Testing patients | **CT-based** | | **Structure-based** | | **Ground truth** | |
| --- | --- | --- | --- | --- | --- | --- |
|  | CN | HI | CN | HI | CN | HI |
| 1 | 0.74 | 0.13 | 0.84 | 0.09 | 0.81 | 0.12 |
| 2 | 0.78 | 0.15 | 0.88 | 0.08 | 0.86 | 0.08 |
| 3 | 0.68 | 0.09 | 0.86 | 0.08 | 0.85 | 0.08 |
| 4 | 0.73 | 0.06 | 0.84 | 0.08 | 0.84 | 0.06 |
| 5 | 0.72 | 0.30 | 0.85 | 0.10 | 0.85 | 0.09 |
| 6 | 0.33 | 0.29 | 0.75 | 0.11 | 0.85 | 0.08 |
| 7 | 0.63 | 0.26 | 0.81 | 0.10 | 0.84 | 0.09 |
| 8 | 0.66 | 0.16 | 0.80 | 0.09 | 0.85 | 0.08 |
| 9 | 0.75 | 0.12 | 0.83 | 0.10 | 0.84 | 0.08 |

CN: conformation number; HI: homogeneity index.
